# Early SARS-CoV-2 Reinfections Involving the Same or Different Genomic Lineages, Spain

**DOI:** 10.3201/eid2906.221696

**Published:** 2023-06

**Authors:** Cristina Rodríguez-Grande, Agustín Estévez, Rosalía Palomino-Cabrera, Andrea Molero-Salinas, Daniel Peñas-Utrilla, Marta Herranz, Amadeo Sanz-Pérez, Luis Alcalá, Cristina Veintimilla, Pilar Catalán, Carolina Martínez-Laperche, Roberto Alonso, Patricia Muñoz, Laura Pérez-Lago, Darío García de Viedma, on behalf of the Gregorio Marañón Microbiology-ID COVID 19 Study Group

**Affiliations:** Gregorio Marañón General University Hospital, Madrid, Spain (C. Rodríguez-Grande, A. Estévez, R. Palomino-Cabrera, A. Molero-Salinas, D. Peñas-Utrilla, M. Herranz, A. Sanz-Pérez, L. Alcalá, C. Veintimilla, P. Catalán, C. Martínez-Laperche, R. Alonso, P. Muñoz, L. Pérez-Lago, D. García de Viedma);; Instituto de Investigación Sanitaria Gregorio Marañón (IiSGM), Madrid (C. Rodríguez-Grande, A. Estévez, R. Palomino-Cabrera, A. Molero-Salinas, D. Peñas-Utrilla, A. Sanz-Pérez, L. Pérez-Lago, D. García de Viedma);; Universidad Complutense, Madrid (R. Alonso, P. Muñoz);; Centro de Investigación Biomédica en Red (CIBER) de Enfermedades Respiratorias-CIBERES, Madrid (R. Alonso, P. Muñoz, D. García de Viedma)

**Keywords:** COVID-19, SARS-CoV-2, respiratory infections, severe acute respiratory syndrome coronavirus 2, SARS, coronavirus disease, zoonoses, viruses, coronavirus, Spain

## Abstract

Centers for Disease Control and Prevention guidelines consider SARS-CoV-2 reinfection when sequential COVID-19 episodes occur >90 days apart. However, genomic diversity acquired over recent COVID-19 waves could mean previous infection provides insufficient cross-protection. We used genomic analysis to assess the percentage of early reinfections in a sample of 26 patients with 2 COVID-19 episodes separated by 20–45 days. Among sampled patients, 11 (42%) had reinfections involving different SARS-CoV-2 variants or subvariants. Another 4 cases were probable reinfections; 3 involved different strains from the same lineage or sublineage. Host genomic analysis confirmed the 2 sequential specimens belonged to the same patient. Among all reinfections, 36.4% involved non-Omicron, then Omicron lineages. Early reinfections showed no specific clinical patterns; 45% were among unvaccinated or incompletely vaccinated persons, 27% were among persons <18 years of age, and 64% of patients had no risk factors. Time between sequential positive SARS-CoV-2 PCRs to consider reinfection should be re-evaluated.

Estimates of the burden of SARS-CoV-2 reinfections continue to be crucial for assessing new SARS-CoV-2 variants with immune escape potential (*1*). Genomic analysis of SARS-CoV-2 strains involved in sequential COVID-19 episodes has been key to assessing the proportion of reinfections, differentiating reinfection from persistent infection, and characterizing reinfection in detail.

Centers for Disease Control and Prevention (CDC) guidelines for consideration of SARS-CoV-2 reinfection require evidence of 2 sequential COVID-19 episodes separated by >90 days and >1 negative RT-PCR in between (*2*). However, inclusion criterion for most studies that have focused on COVID-19 reinfection have usually required 45–60 days between sequential episodes (*3*,*4*). This timeframe maximizes factors that increase the likelihood of reinfection, including the chance of cure of the first episode, clearance of the strain involved in the first episode, and possibility of reexposure to another positive case. Following this philosophy, we reported a systematic population-based analysis of reinfections during the first, second, and third pandemic waves in Spain (*5*). Some studies conducted during Omicron waves described an increase in the proportion of reinfections (*6*,*7*) and a shorter interval between reinfection episodes, such as early reinfections in <60 days. In this study, we aimed to evaluate the possibility of finding reinfections when they are even less likely, <45 days between episodes, and assess which SARS-CoV-2 variants were involved. The study was based on the 15,794 COVID-19 cases diagnosed during November 26, 2021–August 21, 2022, at Gregorio Marañón General University Hospital, a tertiary hospital that serves 650,000 inhabitants in the population of Madrid, Spain. 

## Material and Methods

### Specimens

We selected all cases with 2 sequential COVID-19 episodes at an interval of 20–45 days by considering the time between the last positive reverse transcription PCR (RT-PCR) specimen in the first episode and the first positive specimen in the second episode. We also requested cases for which >1 positive specimen was available in our stored collection, among those taken in the first 10 days of each sequential episode, and for which the specimens had sufficient viral load (cycle threshold [Ct] <32) to maximize the chance of obtaining optimal coverage in whole-genome sequence analysis. To minimize the possibility of including potentially persistent cases, we excluded cases that had clinical conditions or admissions to hospital services that likely corresponded to immunocompromised status.

We used remnants of nasopharyngeal swab specimens previously used for diagnostic purposes via TaqPath COVID-19 CE-IVD RT-PCR kit (ThermoFisher Scientific, https://www.thermofisher.com) during November 26, 2021–August 21, 2022. We extracted viral RNA from nasopharyngeal exudates by using the KingFisher instrument (ThermoFisher Scientific). We used 16 μL of RNA as a template for reverse transcription by using LunaScript RT SuperMix Kit (New England Biolabs, https://www.neb.com).

### Whole-Genome Sequencing

We performed whole-genome amplification of SARS-CoV-2 (Appendix). We deposited sequences above the GISAID quality thresholds into the GISAID database (https://www.gisaid.org); we submitted sequences below the GISAID threshold to the European Nucleotide archive (https://www.ebi.ac.uk/ena; project no. PRJEB56460) (Appendix Tables 1–3). 

**Table 1:** Clinical characteristics of patients with early SARS-CoV-2 reinfection involving the same or different genomic lineages, Spain*

Characteristics	First episode, n = 11	Second episode, n = 11
Average age, y (range)	43.27 (8–88)	43.27 (8–88)
Sex		
M	4 (36.36)	4 (36.36)
F	7 (63.64)	7 (63.64)
Illness severity		
Asymptomatic	4 (36.36)	5 (45.45)
Mild	5 (45.45)	6 (54.55)
Intermediate	2 (18.18)	0
Severe	0	0
Care required		
Emergency	0	0
Hospital admission	3 (27.27)	2 (18.18)
Hospital admission for COVID-19	2 (18.18)	0
Nosocomial transmission	1 (9.09)	0
ICU	0	0
ICU for COVID-19	0	0
Underlying conditions		
None of interest	7 (63.64)	7 (63.64)
High blood pressure	3 (27.27)	3 (27.27)
COPD	1 (9.09)	1 (9.09)
Asthma	0	0
Diabetes	2 (18.18)	2 (18.18)
Ictus	2 (18.18)	2 (18.18)
Overweight/obesity	1 (9.09)	1 (9.09)
Heart disease	2 (18.18)	2 (18.18)
Autoimmune	1 (9.09)	1 (9.09)
Oncological	0	0
Chronic kidney disease	1 (9.09)	1 (9.09)
HIV infection	0	0
AIDS	0	0
Pregnant	0	0
Paxlovid use‡	0	0
Use of dexamethasone	0	0
Death	0	0
Vaccines and serology		
Complete vaccination schedule	6 (54.55)	6 (54.55)
Incomplete vaccination schedule	1 (9.09)	1 (9.09)
Unvaccinated	4 (36.36)	4 (36.36)
Previous positive serology for SARS-CoV-2	0	0
Previous negative serology for SARS-CoV-2	2 (18.18)	2 (18.18)
Serology not available	9 (81.82)	9 (81.82)

*Values are no. (%) except as indicated. COPD, chronic obstructive pulmonary disease; ICU, intensive care unit. †Illness severity was defined according to the following criteria: mild, general unrest, cough, diarrhea, cephalgia, fever, anosmia, myalgias, rhinorrhea; moderate, previous symptoms plus dyspnea, mild respiratory failure, or unilateral pneumonia; severe, previously listed symptoms plus bilateral pneumonia or severe respiratory failure. ‡Nirmatrelvir/ritonavir.

**Table 3:** Clinical characteristics of cases without short-term SARS-CoV-2 persistence in a study of early SARS-CoV-2 reinfection involving the same or different genomic lineages, Spain*

Characteristic	Value, n = 11
Average age, y (range)	58.5 (1–94)
Sex	
M	4 (36.4)
F	7 (63.6)
Illness severity	
Asymptomatic	2 (18.2)
Mild	4 (36.4)
Intermediate	2 (18.2)
Severe	2 (18.2)
Care required	
Emergency	1 (9.1)
Hospital admission	6 (54.5)
Hospital admission for COVID-19	3 (27.3)
Nosocomial transmission	1 (9.1)
ICU	0
ICU for COVID-19	0
Underlying conditions	
None of interest	3 (27.3)
High blood pressure	6 (54.5)
COPD	2 (18.2)
Asthma	1 (9.1)
Diabetes	0
Ictus	1 (9.1)
Overweight/obesity	6 (54.5)
Heart disease	5 (45.5)
Autoimmune	3 (27.3)
Oncological	2 (18.2)
Chronic kidney disease	2 (18.2)
HIV infection	1 (9.1)
AIDS	0
Pregnant	0
Paxlovid use‡	1 (9.1)
Remdesivir use	3 (27.3)
Tocilizumab use	1 (9.1)
Dexamethasone use	4 (36.4)
Death	1 (9.1)
Vaccines and serology	
Complete vaccination schedule	8 (72.7)
Incomplete vaccination schedule	1 (9.1)
Unvaccinated	2 (18.2)
Previous positive serology for SARS-CoV-2	3 (27.3)
Previous negative serology for SARS-CoV-2	2 (18.2)
Serology not available	6 (54.5)

*Values are no. (%) except as indicated. COPD, chronic obstructive pulmonary disease; ICU, intensive care unit. †Illness severity was defined according to the following criteria: mild, general unrest, cough, diarrhea, cephalgia, fever, anosmia, myalgias, rhinorrhea; moderate, previous symptoms plus dyspnea, mild respiratory failure, or unilateral pneumonia; severe, previously listed symptoms plus bilateral pneumonia or severe respiratory failure. ‡Nirmatrelvir/ritonavir.

We considered a case to be a reinfection if different lineages or sublineages were involved in each sequential episode. We also assigned cases as probable reinfections when the sequential strains belonged to the same lineage or sublineage and the sequential strains harbored specific single-nucleotide variants (SNVs) not shared between the first and second episode, indicating that the sequence from the second episode was not derived from the first episode.

### Minority Variant Analysis 

We assessed whether the strain involved in the first episode persisted as a minority variant (i.e., trace of the virus) in the second episode. In each early reinfection case, we used Integrative Genomics Viewer version 2.14.1 (Broad Institute, https://www.broadinstitute.org) to visually inspected SNV alleles called in the strain involved in the first episode in the sequences obtained from the strains involved in the second episode. 

### Short Tandem Repeat Analysis 

We conducted short tandem repeat analysis to ensure that the tested specimens from sequential episodes of all reinfection and probable reinfection cases belonged to the same patient. We used the Mentype Chimera PCR Amplification Kit (Biotype, https://www.biotype.de) to examine 12 noncoding short tandem repeat loci and the sex-specific amylogenic locus on specimens (Appendix).

## Results

The first Omicron variant in our study population was identified during late November 2021. Delta and Omicron variants coexisted during November 26, 2021–January 15, 2022. The study population yielded 66 (0.42%) cases with 2 sequential COVID-19 cases that fulfilled our criteria (Figure). From this initial selection, we excluded 23 cases with clinical conditions or hospitalizations that likely corresponded to an immunocompromised status to minimize the inclusion of potentially persistent cases. Of the remaining 43 cases, 29 had positive specimens in our stored collection representative of 2 sequential episodes that could be analyzed by WGS. For 26 cases (89.7%), we obtained sequences of optimal quality and good coverage from 2 sequential episodes that enabled us to perform a one-to-one genomic comparison of both sequences (Figure).

**Figure:**
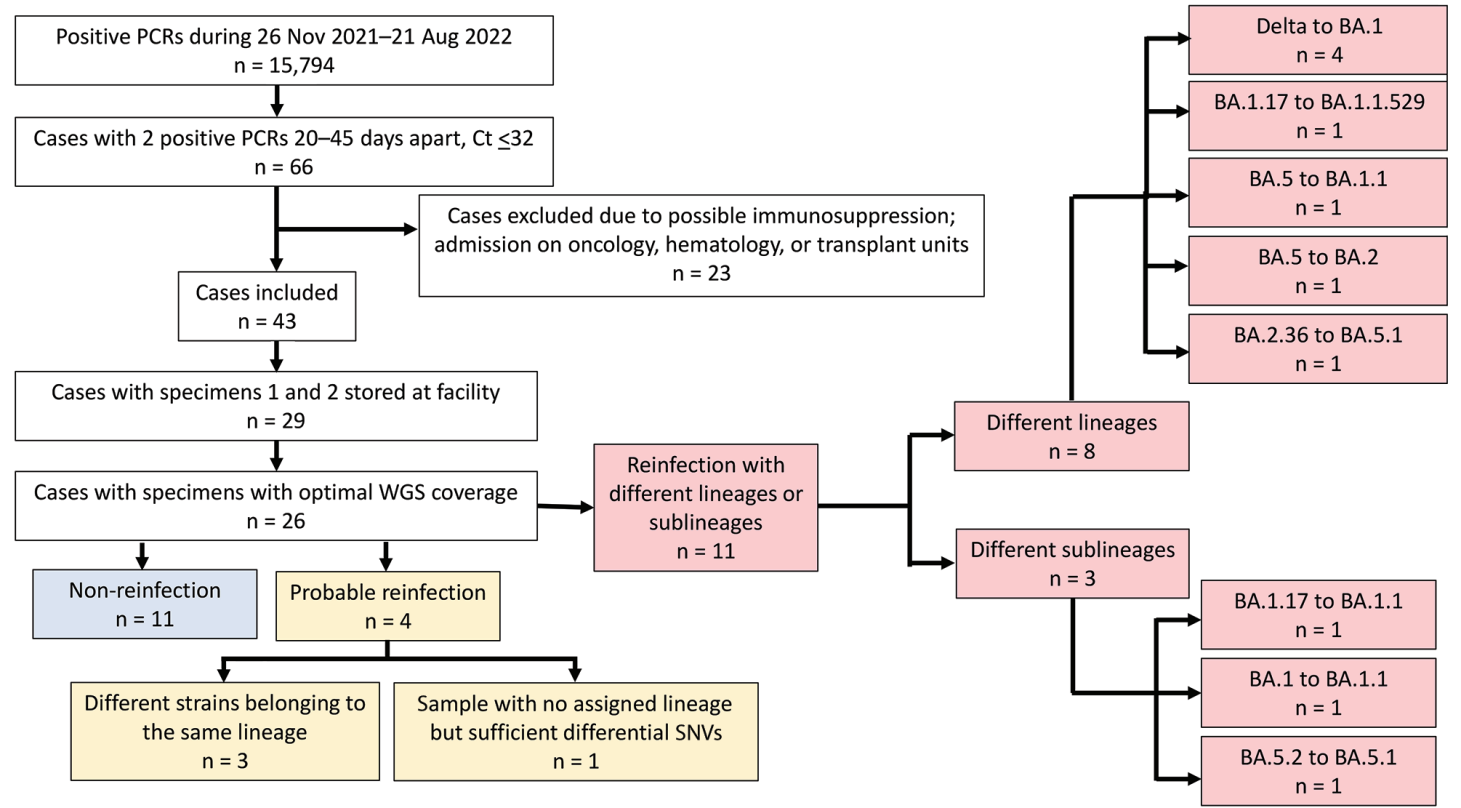
Flowchart of case selection in a study of early SARS-CoV-2 reinfection involving the same or different genomic lineages, Spain. PCR-positive cases were diagnosed by our tertiary hospital, which covers 650,000 inhabitants in the population of Madrid. Among 26 cases with optimal coverage for WGS, 11 were reinfections (red boxes), 4 of which were non-Omicron to Omicron lineage reinfections. Probable reinfection cases (yellow boxes; patients 23–26) showed enough unique SNV differences between the sequences from their sequential specimens to be suspect of reinfection (Appendix Table 3). Ct, cycle threshold; SNV, single-nucleotide variants; WGS, whole-genome sequencing.

In 11 (42%) of the 26 cases, genomic analysis indicated that reinfection occurred and involved different lineages or sublineages in each episode (Figure). Among those 11 cases, 4 involved non-Omicron followed by Omicron variants (i.e., Delta to Omicron BA.1); 4 involved 2 different Omicron lineages (BA.1.17 to B.1.1.529, BA.5 to BA.1.1, BA.5 to BA.2, and BA.2.36 to BA.5.1); and 3 involved different Omicron sublineages (BA.1.17 and BA.1.1, 10 differential SNVs; BA.1 and BA.1.1, 8 SNVs; and BA.5.2 and BA.5.1, 13 SNVs).

We considered another 4 cases to be probable reinfections (Figure): 3 involved different strains from the same sublineage (BA.2, BA.1.1, and BA.1.17); in the fourth case (case 26), we were unable to assign the variant in 1 of the specimens. In all 4 cases, we observed differential SNVs (4–8 SNVs) between the sequences from the sequential episodes. All the differential SNV calls between the sequential episodes were robust, as indicated by the good sequencing coverage (73–2,847 nt depth) observed in those positions (Appendix Table 3). The distribution of the differential SNVs between the sequential sequences in these cases pointed to independent evolutionary pathways; 1–4 SNVs in the first episode were absent in the second, and 3–5 SNVs in the second episode were absent in the first. Those observations ruled out the possibility that the sequence from the second episode evolved from the first sequence, thus indicating that 2 unrelated strains were involved in each of the sequential episodes.

Because of the short time between COVID-19 episodes in our study, we assessed whether the strain involved in the first episode of early reinfected cases could still be traced as remnant minority variants in the second episode. A thorough visual review of SNVs called in the second episode did not identify any minority calls corresponding to SNVs identified in the first episode strain, which indicated that the strain involved in the first episode had been cleared by the time the second infection was established.

We further refined the characterization of reinfections by also performing host genomic characterization to clean up any laboratory errors and ensure that the sequential specimens belonged to the same patient. We performed short tandem repeat analysis on specimens from 15 of 16 cases assigned as reinfections or probable reinfections. For all 15 cases, host genetic analysis confirmed that the 2 sequential specimens used in the study belonged to the same patient. For the remaining 1 case (case 10), no host material was available.

A review of the clinical characteristics of the 11 cases of early SARS-CoV-2 reinfections did not suggest a specific pattern: 63.6% were among female patients, patient ages were 8–88 years, 36.4% of patients had not been vaccinated, and 9.1% had incomplete vaccination schedules (Table 1). Among the unvaccinated case-patients, most were young (8–29 years of age). In most (54.5%) reinfections, symptoms were mild, and 5 patients were asymptomatic. Relevant risk factors were high blood pressure (27.3%), heart disease (18.2%), diabetes (18.2%), and previous ictus (18.3%). In 50% of cases, a SARS-CoV-2 RT-PCR was requested before a procedure or intervention at the hospital or after exposure to a COVID-19 case (Table 2). SARS-CoV-2 antibody serology testing was not available before the first episode for all but 2 cases (cases 10 and 11), but in those cases, serologic results were negative. None of the patients died.

**Table 2:** Clinical characteristics of patients with early SARS-CoV-2 reinfection or probable reinfection involving the same or different genomic lineages, Spain*

Pt. no.	Age, y/sex	Underlying conditions	Illness severity, 1st/2nd episode†	COVID-19 care required, 1st/ 2nd episode	COVID-19 treatment	Vaccine schedule	Inter-infection period, d	PCR Ct, 1st/2nd episode	Reason for PCR, 1st/ 2nd episode	SARS-CoV-2 variant, 1st/2nd episode
Reinfections										
1	29/M	None	Mild/mild	N/N	N	N	37	24/22	Symp/symp	AY.127/BA.1.1.1
2	12/M	None	Mild/mild	N/N	N	N	34	30/19	Symp/symp	AY.124/BA.1.1
3	8/F	None	Asymp/mild	N/N	N	N	37	30/22	PE/symp	B.1.617.2 Delta plus/BA.1.1
4	85/F	HBP, DM, obesity	Asymp/mild	N/N	N	Complete, Pfizer	41	32/25	PP/PE	BA.1.17/BA.1.1
5	27/F	None	Mild/asymp	N/N	N	Complete, AstraZeneca/Pfizer	42	22/32	PP/PP	BA.1.17/B.1.1.529
6	28/F	None	Asymp/mild	N/N	N	Complete, Pfizer/ Moderna	27	32/16	PE/symp	BA.1/BA.1.1
7	42/F	None	Mild/mild	N/N	N	Incomplete, Pfizer	41	32/20	Symp/symp	AY.122/BA.1.17
8	11/F	None	Asymp/ asymp	N/N	N	N	22	30/27	PP/PP	BA.2.36/BA.5.1
9	88/M	COPD, ictus, heart disease, CKD	Mod/asymp	Hospital admission/N	Steroids	Complete, Pfizer	25	13/26	Symp/PP	BA.5/BA.2
10	63/F	HBP, systemic sclerosis	Mild/asymp	N/N	N	Incomplete, AstraZeneca	20	19/32	Symp/PP	BA.5/BA.1.1
11	83/M	HBP, DM, ictus, heart disease	Mod/asymp	Hospital admission/N	Steroids	Complete, Pfizer	27	16/32	Symp/PP	BA.5.2/BA.5.1
Probable reinfections										
23	74/F	HBP, DM, heart disease	NA/NA	NA/NA	NA	Complete, Pfizer	21	31/16	NA/NA	BA.2/BA.2
24	81/M	HBP, DM, heart disease, CKD	Mod/asymp	Hospital admission/ hospital admission	Steroids	Complete, Pfizer	45	22/30	Symp/PP	BA.1.1/BA.1.1
25	64/F	HBP, CKD	Mild/asymp	Emergency/N	N	Incomplete, Pfizer	26	32/30	Symp/symp	BA.1.17/BA.1.17
26	58/M	HBP, DM	Asymp/ asymp	N/N	N	Complete, Pfizer	24	29/30	PP/PP	BA.2/unassigned

*Asymp, asymptomatic; CKD, chronic kidney disease; COPD, chronic obstructive pulmonary disease; DM, diabetes mellitus; HBP, high blood pressure; ICU, intensive care unit; NA, not available; PP, pre-procedure; PE, postexposure; symp, symptoms. †Severity of illness was defined according to the following criteria: mild, general unrest, cough, diarrhea, headache, fever, anosmia, dysgeusia, myalgia, rhinorrhea; Moderate, the above symptoms plus dyspnea, mild respiratory failure, or unilateral pneumonia; Severe, the above symptoms plus bilateral pneumonia or unilateral pneumonia with respiratory failure.

Among the 4 probable reinfections, patient ages were 58–81 years, 2 were male, and 3 had a full vaccination schedule before the first COVID-19 episode (Table 2). Three of the probable reinfections were asymptomatic, but we have no clinical information regarding the COVID-19 episode in the fourth patient. Relevant risk factors were high blood pressure (100%), diabetes (75%), chronic kidney disease (50%), and heart disease (50%).

Another 11 cases in the analysis were not reinfections but short-term persistence involving the same strain. The evolution of the Ct values in those cases was consistent with persistence because most (82%) had higher Ct in the second specimen; 2 had Ct values that were not markedly lower, 3 and 8 cycles difference. The strains corresponded to the Omicron variant and either had acquired no diversity, had 0 SNVs between sequential isolates, or had 1–5 SNVs in the second specimen, consistent with an acquisition of diversity by microevolution during the persistence period. We also reviewed the clinical charts for those case-patients (Table 3); their ages were 1–94 years and 63.6% were female. The most prevalent risk factors were high blood pressure (54.5%), overweight or obesity (54.5%), heart disease (45.5%), and autoimmune diseases (27.3%). Compared with patients who had short-term SARS-CoV-2 persistence, early reinfected patients were younger (43.3 vs. 58.5 years) and had lower baseline pathology (36.4% vs. 72.7%). In terms of clinical severity, 36.4% of patients with early reinfection were asymptomatic in the first episode and 45.5% were asymptomatic in the second episode, compared with only 18.2% of case-patients who had short-term persistence. For the early reinfection group, despite being statistically nonsignificant, the second episode tended to be less severe; in only 3 cases, the second episode was more severe than the first. Among early reinfections, 18.2% of case-patients required hospital admission for COVID-19 during the first episode and none required hospitalization for the second episodes, compared with 27.3% of patients with short-term persistence who required hospitalization. One (9.1%) patient in the short-term persistence group died due to COVID-19 versus none in the early reinfection group.

## Discussion

Most studies focusing on COVID-19 reinfections followed the CDC guidelines during the first waves of the pandemic (*8*). Nevertheless, the guidelines need to be reviewed in the current epidemiologic context, which is substantially different from when most reinfection studies were conducted. One crucial difference is the emergence of the Omicron variant at the end of 2021. Omicron is markedly different from previous variants, harboring a constellation of >55 mutations, 32 of which are in the spike, and 15 mutations map to the receptor-binding domain. Those mutations triggered alarm about the possibility of immune escape from the protection conferred by pre-Omicron variant infections. Those suspicions were confirmed, and Omicron was shown to be barely neutralized by serum from convalescent patients (*9*).

The lack of Omicron neutralization during in vitro exposure to serum from vaccinated or convalescent case-patients infected with earlier variants led to consideration that reinfections were likely to increase. A large study in South Africa demonstrated that risk assessments for reinfection with Omicron were higher than for pre-Omicron variants (*7*). Similarly, the 6.8% reinfection rate with Omicron in Marseille, France, was markedly higher than infection rates (0.2%–1.5%) in pre-Omicron pandemic waves (*6*).

If Omicron escapes the protection associated with infection from earlier variants, then higher rates of Omicron reinfection could be expected to occur within a shorter time after the first episode (i.e., early reinfections) than was seen with previous variants. This shorter reinfection time was noted in Italy (*10*), where Omicron reinfections occurred 25–60 days after the first COVID-19 episode involving the Delta variant, whereas reinfections involving Omicron in both sequential episodes (BA.1 to BA.2) were identified within the standard time range for reinfection, >90 days. Likewise, in Belgium, most early reinfections (<60 days) identified involved Omicron after a Delta infection (*11*). Other studies have also reported shorter times (24 days and 39 days) between episodes involving non-Omicron to Omicron reinfections (*11*,*12*).

To identify early non-Omicron followed by Omicron infections and classify variants as Omicron or non-Omicron, many previous studies relied on indirect inference methods, not WGS. In one study, spike gene target failure, which could be detected in Delta but not Omicron in the TaqPath RT-PCR, was used as a proxy marker to assign the variant (*11*). In another study, variants of concern (VOCs) were inferred by determining changes in the melting patterns of probes used in RT-PCR to target regions where marker SNVs are located (*4*). Although such inferences are useful and practicable, they can only assign reinfections involving certain VOCs, thereby missing possible early reinfections involving the same lineages or even sublineages, which can only be addressed by WGS characterization.

In our study, we tried to optimize the characterization of early reinfections in the Omicron era by performing WGS to cover all possible variants involved, narrowing the time range between episodes to <45 days to capture the earliest reinfections, and fine-tuning the analysis as much as possible by host genetic analysis to ensure that the 2 sequential specimens used for genomic viral comparison belonged to the same patient. During the study period, we detected a total of 66 (0.42%) cases with sequential RT-PCR–positive specimens in an interval of 20–45 days. That percentage was higher than the cases with sequential positives 45–90 days (8 cases, 0.05%) or >90 days (38 cases, 0.24%) apart.

One relevant finding was that among suspected cases of early reinfection, we confirmed early reinfection in 38% (11/29) of cases with specimens available for sequencing. In addition, the time interval between episodes was very short, 20–42 days. A recent systematic review on SARS-CoV-2 reinfections also determined a period of 23–57 days for reinfections (*8*), below the standard 90-day threshold, despite including data from studies published before May 22, 2022; data from the latest waves were also probably underrepresented. More recent criteria for considering reinfections enable reduction to >45 days between episodes for persons with symptoms, evidence of close contact with a confirmed case, and no evidence of other causes of infection (*2*). Our data indicate that even those updated guidelines would miss the early reinfections that we highlight, and these combined findings should lead to reconsideration of the more stringent and longer period of >90 days between episodes used in the CDC guidelines.

About one third (36.4%) of the early reinfections in our study involved sequential infection with non-Omicron followed by Omicron variants, which is consistent with previous descriptions of Omicron variants capable of causing immediate reinfection of patients newly recovered from COVID-19 (*9*). However, because of our nontargeted WGS-based design, we were able to identify not only early reinfections involving non-Omicron followed by Omicron variants but also reinfections with different Omicron lineages, different sublineages belonging to the same Omicron lineage, and even different strains from the same sublineage that were missed in other studies that used indirect inference methods. Our findings support reformulating the assumption that early infections are mainly restricted to a non-Omicron–Omicron alternation, because of the lack of cross-protection caused by major Omicron genetic differences. 

Among the 4 probable early reinfections in our study, 3 cases involved 2 strains from the same sublineage. One case (case 23) constituted one of the shortest time intervals between episodes, 21 days apart, which contrasts with other studies that only found reinfections with the same variant for episodes >90 days apart. This probable early reinfection showed 8 SNVs between strains from the same lineage in the 2 separate episodes. In addition, several observations led us to reinforce its assignment as an early reinfection. First, the patient had 3 RT-PCR–negative specimens between the 2 RT-PCR–positive specimens 21 days apart, which sustains the hypothesis of early reinfection versus the alternative explanation of persistence. Second, the Ct value of the second specimen was 16, whereas the Ct of the first specimen was 31. We generally expect an increased Ct value, or reduced viral load, for a second specimen in cases of persistence, but a new reinfection should correspond to a lower Ct value, as noted in that case. Third, for persistence we expect a sequential acquisition of SNVs from the first strain during the persistence period. To the contrary, in that case, when we analyzed the distribution of the 8 SNVs identified between the 2 sequential specimens, 4 SNVs were only identified in the first specimen and another 4 were identified only in the second specimen, which is more consistent with the involvement of 2 independent strains, each with 4 proper SNVs.

The robustness of our assignation of early reinfections is supported by the precautionary consideration of the possibility of specimens belonging to different persons could be mishandled or misclassified, thereby leading to erroneous assignment as reinfections (*13*). However, we confirmed the hosts in all our reinfections by performing host analysis. Most of the literature focused on COVID-19 reinfections, with just a few exceptions (*5*,*13*,*14*), lacks host control.

We identified no common clinical pattern among early reinfection cases by sex, age, risk factors, or clinical conditions. Although we did not achieve strong statistical support because of our small sample size, we observed a tendency for the second episode in early reinfections to be equally or less severe than the previous episode. Of note, more than half (63.6%) of the reinfections were cases with no clinical history, which means that we need to broaden the circumstances for suspecting early reinfections.

In our analysis, despite the efforts to minimize the interference of persistence in case selection by ruling out cases with positive PCRs between episodes and patients with immunosuppression, we still identified 11 cases in which the same strain was found in the 2 sequential episodes, even though 27.3% of those cases had no clinical history to justify persistence. Although those were cases of short-term persistence, our findings could help expand clinical patterns to consider unexpected persistence, which is different from long-term persistence that occurs mainly in immunosuppressed persons (*15*,*16*). Despite the short-term nature of such persistence, the findings could still be relevant, depending on clinical interpretations and isolation measures. 

Our data fill a gap in observations of the time range between sequential COVID-19 episodes that has generally been missing from the literature. In addition, our study period covered the 6th, most recent, COVID-19 wave, to provide new information on reinfections in a scenario in which SARS-CoV-2 VOCs are emerging and the population has extensive vaccine coverage. To reinforce the robustness of our findings, we also provided additional analytical rigor and refinement by including host genetic analysis in the assignment of reinfection.

In conclusion, our study provides new data on early reinfections involving Omicron and other variants. These findings shorten the time between episodes in which reinfection can occur and broaden the clinical profile for reinfection beyond unvaccinated young persons. We showed that early reinfections are not exclusively associated with the impaired protection expected of a non-Omicron to Omicron sequence but also can involve very similar strains. Because early reinfection can occur in various clinical and epidemiologic circumstances, guidelines for assigning reinfection to only >90 days between sequential SARS-CoV-2–positive PCRs should be reevaluated.

Appendix Additional information on early SARS-CoV-2 reinfection involving the same or different genomic lineages, Spain.
