# Supplementary material for: Early SARS-CoV-2 Reinfections Involving the Same or Different Genomic Lineages, Spain
Source: Emerg Infect Dis. 2023 Jun;29(6):1154–61. doi: 10.3201/eid2906.221696 (PMC10202887; doi:10.3201/eid2906.221696)
Supplement: Appendix — Additional information on early SARS-CoV-2 reinfection involving the same or different genomic lineages, Spain. [file 22-1696-Techapp-s1.pdf]

Article DOI: https://doi.org/10.3201/eid2906.221696

EID cannot ensure accessibility for supplementary materials supplied by authors. Readers who have difficulty accessing supplementary content should contact the authors for assistance.

Early SARS-CoV-2 Reinfections Involving the Same or Different Genomic Lineages, Spain

Appendix

Whole-Genome Sequencing

Whole genome amplification of SARS-CoV-2 was done with Artic_nCov-2019_V4 and V4.1 primer panels (Integrated DNA Technologies, Inc., https://www.idtdna.com) (https://artic.network/ncov-2019) and Q5 Hot Start DNA polymerase (New England Biolabs, https:// www.neb.com). Libraries were prepared using the Nextera DNA Flex Library Prep Kit (Illumina, https://www.illumina.com) and sequenced in pools on MiSeq equipment (2 × 150 bp).

Sequences above the GISAID quality thresholds were deposited in GISAID (https://www.gisaid.org) and those below the GISAID thresholds were submitted to ENA (PRJEB56460) (Appendix Table 1). An in-house analysis pipeline was applied to the sequencing reads (https://github.com/MG-IiSGM/covid_multianalysis). In brief, the pipeline involves the following 4 steps: 1) pre-processing and quality assessment of fastq files using fastp version 0.20.1 (https://doi.org/10.1093/bioinformatics/bty560) and fastQC version 0.11.9 (https://www.bioinformatics.babraham.ac.uk/projects/fastqc); 2) mapping with BWA version 0.7.17 (https://arxiv.org/abs/1303.3997) and variant calling using iVar version 1.3.1 (https://doi.org/10.1186/s13059-018-1618-7); the wild type SARS-CoV-2 sequence (GenBank accession no. NC_045512.2) was used as reference; 3) genome annotation with SnpEff version 5.1 (https://doi.org/10.4161/fly.19695) and wild type SARS-CoV-2 as reference, and lineage designation with Pangolin version 4.0.2 (https://github.com/cov-lineages/pangolin); and 4) calibration of occasional low coverage positions using joint variant calling.

Short Tandem Repeat Analysis

Short Tandem Repeat (STR) analysis was applied for human identity testing and to ensure that the tested specimens from sequential episodes of reinfection cases belonged to the same patient. The Mentype Chimera PCR amplification kit (Biotype, Germany) was used on the specimens used for SARS-CoV-2 genome sequencing. We examined 12 non-coding STR loci and the sex-specific amelogenin locus, labeled with 3 different dyes (6-FAM, BTG, or BTY). PCR was performed with 0.2–1 ng of genomic DNA using the Mentype Chimera PCR amplification kit (Biotype, Germany), the GeneAmp PCR System 9700 Thermal Cycler, followed by capillary electrophoresis on the 3030x1Genetic Analyzer (ThermoFisher Scientific, MA, USA), according to the manufacturers' instructions.

Appendix Table 1. Sequences deposited in GISAID and ENA from a study of early SARS-CoV-2 reinfection involving the same or different genomic lineages, Spain*

Patient no.	Episode no.	GISAID/ENA ID
1	1	EPI_ISL_15213997
	2	EPI_ISL_15214010
2	1	EPI_ISL_15214032
	2	EPI_ISL_15214024
3	1	ERR10307499
	2	EPI_ISL_15214011
4	1	EPI_ISL_15214044
	2	EPI_ISL_15214009
5	1	EPI_ISL_15214013
	2	EPI_ISL_15214025
6	1	EPI_ISL_15214029
	2	EPI_ISL_15214020
7	1	ERR10307501
	2	EPI_ISL_15214014
8	1	EPI_ISL_15214004
	2	EPI_ISL_13577683
9	1	EPI_ISL_15214039
	2	EPI_ISL_15214027
10	1	EPI_ISL_15214031
	2	EPI_ISL_15214018
11	1	EPI_ISL_15214008
	2	EPI_ISL_15214033
12	1	EPI_ISL_8306944
	2	EPI_ISL_15214012
13	1	EPI_ISL_15214045
	2	EPI_ISL_15214019
14	1	EPI_ISL_8926943
	2	EPI_ISL_15214042
15	1	EPI_ISL_13476995
	2	EPI_ISL_13476918
16	1	EPI_ISL_15214002
	2	EPI_ISL_15214037
17	1	EPI_ISL_12687954
	2	EPI_ISL_15214026
18	1	EPI_ISL_12687959
	2	EPI_ISL_15214043
19	1	EPI_ISL_15214015
	2	EPI_ISL_15214016
20	1	EPI_ISL_15214022
	2	EPI_ISL_15214035
21	1	EPI_ISL_15214023
	2	EPI_ISL_15214036
22	1	EPI_ISL_15214007
	2	EPI_ISL_15214030
23	1	EPI_ISL_12688015
	2	EPI_ISL_15214001
24	1	EPI_ISL_13638547
	2	EPI_ISL_15214034
25	1	EPI_ISL_15214028
	2	EPI_ISL_15214021
26	1	EPI_ISL_15214006
	2	ERR10307503

*Sequences deposited in GISAID (https://www.gisaid.org) and ENA (https://www.ebi.ac.uk/ena). Patients 1–11 had early reinfections; patients 12–22 did not have reinfections (short-term persistence); patients 23–26 had probable reinfections.

Appendix Table 2. Sequencing quality values of SARS-CoV-2 in a study of early reinfection involving the same or different genomic lineages, Spain

Patient no.	Specimen no.	Coverage >30×	Result
1	1	98.20	Reinfection
	2	99.55	Reinfection
2	1	52.55	Reinfection
	2	86.95	Reinfection
3	1	38.84	Reinfection
	2	99.56	Reinfection
4	1	93.99	Reinfection
	2	98.98	Reinfection
5	1	99.30	Reinfection
	2	82.90	Reinfection
6	1	65.46	Reinfection
	2	99.56	Reinfection
7	1	47.03	Reinfection
	2	99.55	Reinfection
8	1	98.10	Reinfection
	2	98.55	Reinfection
9	1	97.18	Reinfection
	2	75.14	Reinfection
10	1	56.01	Reinfection
	2	99.48	Reinfection
11	1	98.53	Reinfection
	2	48.80	Reinfection
12	1	96.91	Short-term persistence
	2	99.53	Short-term persistence
13	1	94.56	Short-term persistence
	2	98.79	Short-term persistence
14	1	98.27	Short-term persistence
	2	96.80	Short-term persistence
15	1	98.73	Short-term persistence
	2	97.31	Short-term persistence
16	1	99.17	Short-term persistence
	2	96.92	Short-term persistence
17	1	94.70	Short-term persistence
	2	46.23	Short-term persistence
18	1	97.19	Short-term persistence
	2	97.31	Short-term persistence
19	1	99.48	Short-term persistence
	2	98.87	Short-term persistence
20	1	99.47	Short-term persistence
	2	97.25	Short-term persistence
21	1	99.48	Short-term persistence
	2	97.90	Short-term persistence
22	1	98.63	Short-term persistence
	2	57.76	Short-term persistence
23	1	99.60	Probable reinfection
	2	98.71	Probable reinfection
24	1	99.58	Probable reinfection
	2	48.28	Probable reinfection
25	1	72.42	Probable reinfection
	2	98.70	Probable reinfection
26	1	99.43	Probable reinfection
	2	28.40	Probable reinfection

Appendix Table 3. Sequencing coverages obtained for the differential SNVs involved in the probable reinfections in a study of early reinfection involving the same or different genomic lineages, Spain*

					Specimen 1				Specimen 2			
Pt no.	Spec. 1	Unique SNVs	Spec. 2	Unique SNVs	POS	ALT_ FREQ	TOTAL_ DP	Gene	POS	ALT_ FREQ	TOTAL_ DP	Gene
23	BA.2	4	BA.2	4	1143	0.9991	2398	ORF1ab	21721	0.8238	477	ORF1ab
					5312	0.8581	2581	ORF1ab	22326	0.9870	155	S
					22458	0.9564	1585	S	22792	0.9098	255	S
					27731	0.9975	2847	ORF7a	25352	0.8310	373	S
24	BA.1.1	1	BA.1.1	5	5386	1.0	724	ORF1ab	13550	0.9791	192	ORF1ab
									22987	0.9753	81	S
									22993	0.9638	83	S
									24917	0.9401	384	S
									27899	0.9860	358	ORFB
25	BA.1.17	1	BA.1.17	3	26916	0.7123	73	M	11282	0.9217	2263	ORF1ab
									21762	0.9970	687	S
									21764	0.9911	1014	S
26	BA.2	2	†	4	17410	0.9990	1072	ORF1ab	868	0.9852	610	ORF1ab
					25584	0.9990	1096	ORF3a	11110	1.0	2204	ORF1ab
									22689	1.0	723	S
									25665	1.0	1174	ORF3a

*DP, coverage depth; FREQ, frequency; ORF, open reading frame; POS, position; Pt, patient; S, spike; SNVs, single nucleotide variants; Spec., specimen.
†Unassigned.
